# Ecofriendly Biopolymer-Based Nanocomposite Films with Improved Photo-Oxidative Resistance

**DOI:** 10.3390/ma15165778

**Published:** 2022-08-21

**Authors:** Elisabetta Morici, Giulia Infurna, Nadka Tz. Dintcheva

**Affiliations:** 1 Dipartimento di Ingegneria, Università degli Studi di Palermo, Viale delle Scienze, Ed. 6, 90128 Palermo, Italy; 2 ATeN Center, Università di Palermo, Viale delle Scienze, Ed. 18, 90128 Palermo, Italy

**Keywords:** layered double hydroxides (LDH), hindered amine light stabilizer (HALS), carrier for moieties, biopolymer nanocomposites, degradation

## Abstract

The interest towards high performance biopolymer-based materials increases continuously and, to guarantee appropriately industrial applications, the photo-oxidative resistance and stability of these materials must be adequately addressed. In this study, innovative biopolymer-based nanocomposites, i.e., Polyamide 11 (PA11), containing ad-hoc modified Layered Double Hydroxides (LDH), were successfully formulated and characterized. Particularly, LDH were considered carriers for hindered amine light stabilizing molecules, so two different hindered amine moieties (HALS1 and HALS2) were anchored on LDH layered internal structures and/or outer surfaces. The presence of HALS1 and HALS2 in LDH were confirmed by X-ray diffraction, spectroscopy, and thermogravimetric analysis. Then, the novel LDH-HALS nanofillers (here named LDH-HALS1 and LDH-HALS2) were introduced into a PA11 matrix by melt mixing at 5 wt.%; the produced nanocomposites were characterized by differential scanning calorimetry, rheological, and morphological analysis. All obtained results suggest that the LDH-HALS1/HALS2 nanofillers were very well dispersed into the PA11 matrix. Additionally, the photo-oxidative resistance of the PA11-based nanocomposite films was evaluated by subjecting thin films to UVB exposure and the degradation process was monitored by spectroscopic analysis over time. The photo-oxidative resistance of the PA11/LDH-HALS1/HALS2 was compared to that of PA11-based nanocomposites containing unmodified LDH and the commercial hindered amine UV-stabilizer (Cyasorb^®^ UV-3853). It was established that by anchoring the hindered amine moieties to the LDH, the PA11 nanocomposites were successfully protected against UVB exposure. This was because the hindered amine light stabilizing molecules were available to act at the critical zone where the degradation phenomena occur, which is at the interface between the matrix and the inorganic particles.

## 1. Introduction

Presently, biopolymer-based nanocomposites continue to receive attention for use as advanced sustainable materials because of their ecofriendly nature, improved nanocomposite properties, and performance. These advantages are due to the presence of a small amount of nanofillers (1–10wt%) [1,2,3,4]. As extensively documented in the last two decades, the adding of nanofillers, such as layered double hydroxides (LDHs), montmorillonite (MMt), silica, wollastonite, halloysite nanotubes, calcium carbonate, carbon nanotubes, fullerenes, graphene nanoplatelets, graphene oxide, metal oxides, and others, significantly enhances the barrier properties, flame retardance, mechanical, and thermomechanical resistance of polymers and biopolymers [1,2,3,4]. Additionally, to improve the nanofillers compatibility and dispersion in polymers and biopolymers, the nanoparticles are appropriately chemically treated and/or organomodified [5,6,7].

**Figure 1:**
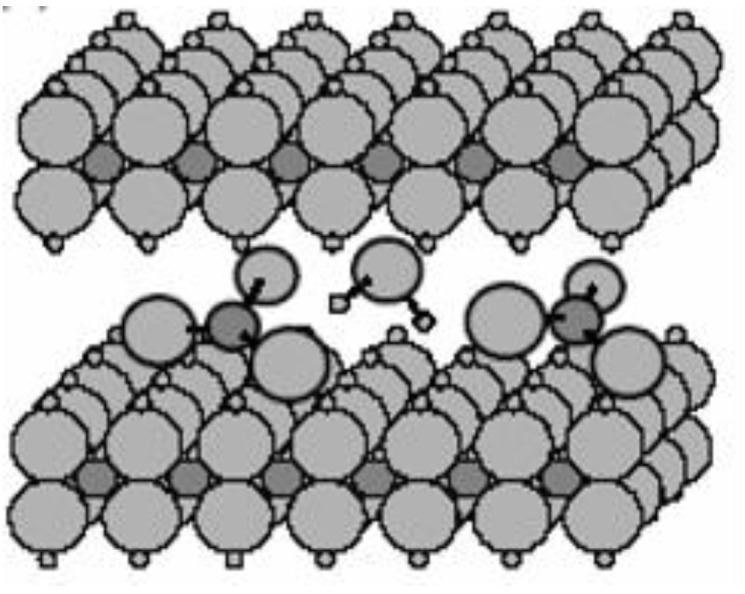
Typical structure of nanoceramics, i.e., metal hydroxide layers and interlayer anions [6].

A typical structure of pristine nanoceramics, such as LDHs or montmorillonite, is shown in Figure 1. Particularly, the general formula of LDHs is [M^2+^_1−__x_M^3+^_x_(OH)_2_](A^m−^)_x/m_·*n*H_2_O, where M^n+^ are metal cations (M^2+^ = Mg^2+^, Zn^2+^, Ni^2+^, Ca^2+^, Cu^2+^, etc., M^3+^ = Al^3+^, Fe^3+^, etc.) and A^m-^ are interlayer anions (A^m-^ = CO_3_^2−^, NO_3_^−^, SO_4_^2−^, etc). The LDHs structure presents as a stacking of brucite Mg(OH)_2_ in which Mg(OH)_6_ octahedra are connected through edge sharing forming 2D sheets with a spacing of 4.8 A. In actuality, some of the M^2+^ in the brucite layers are substituted with M^3+^ developing a permanent cation layer charge. In the interlayer space, charge-balancing anions and water molecules attracted through hydrogen bonding are located, and they are simply exchangeable. Therefore, the LDHs are widely applicable as carriers for various supramolecular structures of heterogeneous hybrid systems because of their noticeable abilities to both absorb organic compounds and exchange anions, except for carbonate anions [8,9,10].

However, although the adding of nanofillers to polymers and biopolymers leads to the formulation of high performance micro-/nano-composite materials, unfortunately, some of these materials show reduced oxidative degradation due to the presence of the nanoparticles [11,12,13,14,15,16,17,18,19]. It is known that polymers, biopolymers, and their micro-/nano- composites undergo thermo- and photo- oxidative degradation during both processing and service life. The prevention of degradation through the introduction of stabilizing molecules, such as antioxidants and light stabilizers, is absolutely imperative for nanocomposite large-scale applications [11,12,13,14]. In some cases, the added nanofillers could accelerate the oxidative degradation of polymers and even more of biopolymer matrices. Unfortunately, this issue limits the applications of nanocomposites on a large industrial scale. According to the literature, the prodegradant effect of some nanoceramics could be explained by considering the presence of some transition metal ions (e.g., iron ions) and/or organic impurities in conjunction with improper dispersion and distribution. These lead to the formation of aggregates in the matrices [15,16]. Moreover, because some stabilizing molecules become entrapped between nanoceramic layers and cause untimely inactivation, added stabilizing systems are not able to offer the expected protection in terms of processing stability and extension of nanocomposite service life [17,18,19].

Additionally, the stabilizing molecules show poor thermal stability and can easily volatize during processing at typical high processing temperatures. This causes them to migrate from the bulk to the surface of manufacts. To avoid these inconveniences, the hindered phenols, phosphites, phosphonites, and hindered amines can be linked with long alkyl chains. This approach was considered at a large industrial scale [8]. Indeed, the latter strategy was a winning choice considering the stabilization of pure polymers and biopolymers, but it is not applicable to the stabilization of nanocomposites because of entrapment and inactivation of the active stabilizing molecules between ceramic layers, as mentioned above.

Interestingly, to improve the stability of antioxidants and light stabilizers, and to avoid the stabilizers becoming entrapped between nanoceramic layers, two different approaches have been currently proposed in the scientific literature: Specifically, the anchoring of the stabilizing molecules through chemical linkage onto particles [6,20,21] and/or on polymer chains [22], and the immobilization of stabilizing molecules through physical adsorption onto the nanoparticles another surface [23,24]. The chemical linkage is usually achieved through the covalent bonding of stabilizing molecules onto organic modifiers of different nanoparticles, such as nanosilica [18] and nanoceramics [25]. The absorption is usually proposed considering that the inorganic particles are an efficient carrier for natural stabilizing molecules. In this way, the stabilizing functionalities are preserved. Therefore, in both cases, the stabilizing molecules are available to exert their action at the interface between inorganic particles and host matrices, which is a critical zone for the beginning of degradation.

In this study, LDH was employed as a carrier for two different hindered amine light stabilizers (herein named HALS1 and HALS2), and then, the obtained nanofillers were introduced into a biopolymer matrix through melt mixing. In this way, the stabilizing molecules can act at the interface between the matrix and inorganic particles, which is the critical zone for the beginning of nanocomposite degradation. The application of this innovative approach to the stabilization of nanocomposites, i.e., the formulation of nanofillers with in-build stabilizing functions, allows for the formulation of nanocomposite films with improved oxidation resistance. The formulated nanocomposites were characterized by differential scanning calorimetry in addition to rheological and morphological analysis, and they were subjected to accelerated UVB exposure to evaluate their photo-oxidative resistance. It was found that by anchoring the hindered amine moieties to the LDH, the nanocomposites were successfully protected against UVB exposure because the HALS molecules were available to act at the interface between the matrix and inorganic particles, which is the critical zone where the degradation phenomena occur.

## 2. Materials and Methods

### 2.1. Materials

The polyamide used in this work was a polyamide 11, PA11, (Nylon 11, pellets form, from Sigma–Aldrich), with a glass transition temperature Tg = 46 °C, melting temperature Tm = 198 °C, and density ρ = 1.026 g/cm^3^ at 25 °C; molecular weight M_w_ = 201.31 g/mol, MFI@235 °C/2.16 kg = 14.5 ± 1.2 g/10 min.

Layered Double Hydroxides (LDH) Pural MG63HT, a commercial magnesium aluminum hydroxy carbonate (LDH-CO_3_), was kindly supplied by Sasol Germany GmbH, with the molecular formula Mg_0.66_Al_0.34_(OH)_2_(CO_3_)_0.17_·0.62H_2_O, as previously documented [P94].

**Scheme 1:**
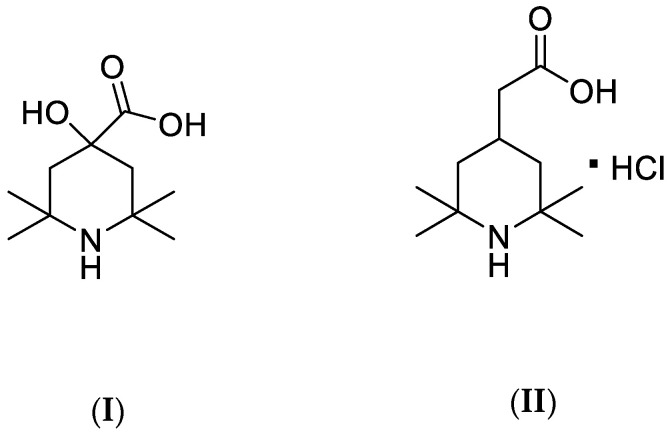

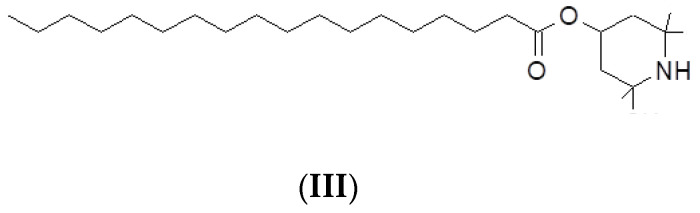
Chemical formula of considered HALS.

The Hindered Amine Light Stabilizers: 4-hydroxy-2,2,6,6-tetramethylpiperidine-4-carboxylic acid, herein named HALS1, see (I) in Scheme 1, and (2,2,6,6-Tetramethylpiperidin-4-yl)acetic acid hydrochloride, herein named HALS2, see (II) in Scheme 1, were purchased from Sigma–Aldrich and used as received, i.e., without further purification.

The Hindered Amine Light Stabilizer, HALS, (UV, molecular weight of 423.72 g/mol; C27H53NO2) is 2,2,6,6-Tetramethyl-4-piperidinyl stearate from Cytec^®^ (Cyasorb^®^ UV-3853), see (III) in Scheme 1.

Methanol (Sigma–Aldrich, St. Louis, MO, USA), chloroform (Carlo Erba, RPE grade, Emmendingen, Germany), and chloroform (Sigma–Aldrich, HPLC grade, ≥99.8%, ethanol stabilized) were used without further purification.

### 2.2. Modification of LDH-CO_3_ to LDH/HALS

To prepare LDH containing HALS molecules, LDH-CO_3_ was converted to nitrate form LDH-NO_3_, according to the titration procedure [5,26]. Specifically, LDH-CO_3_ was dispersed in a 1 M NaNO_3_ aqueous solution (mass/volume = 2 g/100 mL), and the suspension was titrated with a 1 M HNO_3_ solution. After titration, the white solid was washed several times with CO_2_-free deionized water and dried overnight at 60 °C in a vacuum oven. The calculated anion-exchange capacity (AEC) of LDH-NO_3_, which has the formula Mg_0.66_Al_0.34_(OH)_2_(NO_3_)_0.34_·0.44H_2_O, is 3.85 mmol of NO_3_^−^/g [26]. The LDH-HALS1/HALS2 were then obtained by anion exchange. An amount of HALS1 and HALS2, corresponding to 1.5 times the AEC of the LDH-NO_3_, was dissolved in 300 mL of CO_2_-free deionized water and heated at 70 °C. Then 1 g of LDH-NO_3_ was added to the solution under a nitrogen atmosphere, and then kept under stirring for three days in dark conditions at 70 °C. Both LDH-HALS1 and LDH-HALS2 were recovered by filtration, washed several times with deionized CO_2_-free water until pH = 7, and finally dried under a vacuum at 60 °C to constant weight.

### 2.3. Processing of the Nanocomposites

The nanocomposites (LDH-NO_3_, LDH-HALS1, and LDH-HALS2 added at 5 wt.%) were processed in a Haake Microconical corotating twin-screw extruder (CTW5). The melt was continuously processed using the integrated bypass valve which allows the recirculation of the melt via the backflow channel, and the entrance and exit pressures, through the slit capillary backflow channel, were measured. Torque and pressure versus time curves were monitored continuously throughout the processing of the neat PA11 and PA11/LDH nanocomposites. All LDH, with and without HALS, were force-fed at 5 wt.% into the polymer melt after 2 min of processing the neat polymer. For PA11 and PA11/LDH -based samples, a flat temperature profile of 210 °C and a speed of 100 rpm were employed.

Thin PA11 and PA11/LDH films (~80 mm thick) were prepared through compression moulding using a Carver Press at: temperature of 210 °C; load of 5000 psi; cooling time of 5 min. Before the moulding, the neat matrix and all the PA11/LDH were dried overnight at 90 °C in a vacuum oven.

### 2.4. Characterizations

A Fourier Transform Infrared Spectrometer (Spectrum One, Perkin Elmer) was used to record IR spectra using 16 scans at a resolution of 1 cm^−1^. Measurements were obtained from the average of triplicate samples with a calculated maximum experimental error (relative standard deviation) of around 5%.

The progress of degradation for neat PA11 and PA11-based systems has been followed by performing FTIR spectra over the time and monitoring the peak height at 1715 cm^−1^ (using Spectrum One software) vs. time, i.e., the peak’s height at given exposure times (h_t_), minus the peak’s height before exposure (h_t0_).

The spectra of LDHs were obtained by mixing the samples with potassium bromide (KBr 99.4% spectroscopic grade purchased from Sigma–Aldrich).

Wide-angle X-ray diffraction (WAXD) analysis was performed at room temperature with a Siemens D-500 Krystalloflex 810 diffractometer used in the reflection mode, with Cu-Kα radiation at λ = 0.1542 nm and a scan rate of 1.0 deg/min; the diffraction spectra were obtained over a 2θ range of 1.5–30°. The interlayer spacing between the LDH layers, d_003_, was computed by applying Bragg’s law.

Thermogravimetric analysis (TGA) was performed using an Exstar TG/DTA Seiko 7200 instrument. Samples (5–10 mg) were placed in alumina sample pans and runs were carried out at the standard rate of 10 °C min^−1^ from 30 to 900 °C under air flow (200 mL min^−1^).

The calorimetric data were evaluated using differential scanning calorimetry (DSC) using a Perkin–Elmer DSC7 calorimeter. All experiments were performed under dry nitrogen on samples of about 10 mg in 40 μL sealed aluminium pans. For neat PA11 and PA11-based nanocomposites, four calorimetric scans (two heating: 30–220 °C and two cooling: 220–30 °C) were performed for each sample at a scanning heating/cooling rate of 5 °C/min. The values of heat flow have been normalized considering the samples’ mass.

Rheological tests were performed using a stress-controlled rheometer (Rheometric Scientific, SR5, TA Instrument, New Castle, DE, USA) in parallel plate geometry (plate diameter 25 mm). The complex viscosity (η*), storage (G′), and loss (G″) moduli were measured under frequency scans from ω = 10^−1^ to 10^2^ rad/s at T = 210 °C for PA11 and PA11/LDH based samples. The strain amplitude was γ = 5%, which preliminary strain sweep experiments proved to be low enough to be in the linear viscoelastic regime.

The nanocomposite structures were further evaluated by qualitatively characterising the degree of dispersion of the LDH’s selected nanocomposite samples using SEM. The SEM analysis was performed on cryogenically fractured and gold sputtered surfaces of thin compression moulded samples using a Philips (Eindhoven, Netherlands) ESEM XL30 scanning electron microscope.

### 2.5. Photo-Oxidation

Ultraviolet (UV) exposure tests were carried out in a QU-V chamber containing eight UV-B lamps–313 nm, (Q-lab Corp, Westlake, CA, USA). The exposure cycle conditions were 8 h of light at T = 55 °C followed by 4 h condensation at T = 35 °C. The progress of photodegradation was followed by FTIR spectroscopy.

## 3. Results and Discussion

### 3.1. Characterization of LDH/HALS

**Figure 2:**
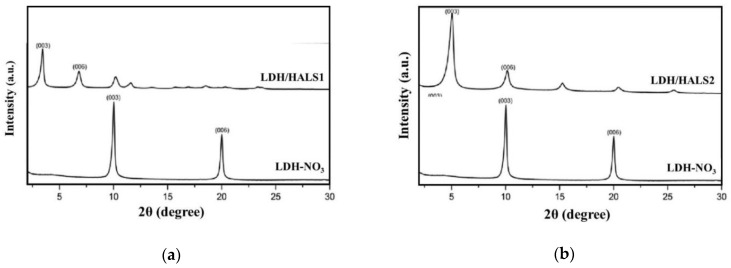
WAXD patterns of (**a**) LDH-HALS1 and (**b**) LDH-HALS2 in comparison to the pattern of LDH-NO_3_.

**Figure 3:**
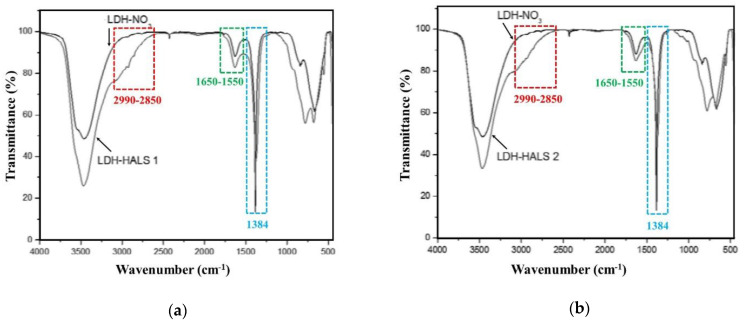
FTIR spectra of (**a**) LDH-HALS1 and (**b**) LDH-HALS2 in comparison to the spectrum of LDH-NO_3._.

**Figure 4:**
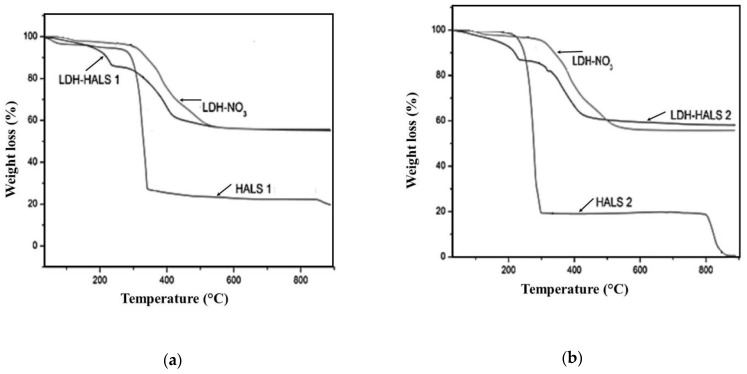
TGA curves of (**a**) LDH-HALS1 and neat HALS1 and (**b**) LDH-HALS2 and neat HALS2 in comparison to the curve of LDH-NO_3_.

Two different LDH/HALS1 and LDH/HALS2 were prepared and subjected to WAXD, FTIR, and TGA analysis, and in Figure 2, Figure 3 and Figure 4 all obtained results, in comparison to that of LDH-NO_3_, are shown. Therefore, Figure 2a,b shows the WAXD patterns of both modified LDH in comparison to that of LDH-NO_3_. According to the literature, the LDH-NO_3_ has a basal spacing of 0.89 nm (reflection at 2θ = 9.9°, corresponding to (003)), [5,26], while new modified LDH have a basal spacing of ca. 2.35 nm (reflection at 2θ = 3.7°) and ca. 1.55 nm (reflection at 2θ = 5.3°). This suggests the occurrence of LDH intercalation due to the presence of HALS1 and HALS2 molecules.

Further confirmation about the presence of HALS1 and HALS2 molecules in LDH structure comes from FTIR analysis, see Figure 3a,b. As expected, due to the presence of HALS molecules, the FTIR spectra of LDH-HALS1 and LDH-HALS2 show different additional absorption bands in comparison to the typical FTIR bands of LDH-NO_3_. Specifically, the peak at ca. 1560 cm^−1^ is assigned to the stretching vibration of the carboxylate groups (C=O(O)-) in both HALS1 and HALS2, and this peak is partially overlapped with the peak centered at 1630 cm^−1^, which is attributed to the bending vibration of crystal water in LDH structure. Broad shoulders/peaks in the range 2990–2850 cm^−1^ in the spectra of both LDH-HALS1 and LDH-HALS2 can be noticed, and they can be assigned to the C-H stretching vibrations of -CH and -CH_3_ groups; therefore, confirming the presence of HALS molecules in LDH. Moreover, the absorption bands at 1384 cm^−1^, due to the stretching vibration of nitrate groups, are clearly visible in the spectra of both LDH-HALS1 and LDH-HALS2, suggesting that the exchange of LDH-NO_3_ by both HALS 1 and HALS2 occurred partially.

In Figure 4a,b, the TGA curves of LDH-HALS1 and LDH-HALS2 are plotted in comparison to the curve of LDH-NO3. The curve of LDH-NO_3_ shows a two-step decomposition process: the first weight loss step (weight loss ca. 2.5%), occurs between 100 and 150 °C due to the loss of adsorbed and intercalated water molecules; the second weight loss step (residual weight ca. 55.6%), occurs between 250 and 700 °C due to dehydroxylation of the metal hydroxide layers and the loss of nitrate ions, also according to the literature [5,26].

It is worth noting that both LDH-HALS1 and LDH-HALS2 show a three-step decomposition process: the first weight loss step (ca. 2.5%) takes place between 100 and 150 °C and it can be attributed to the loss of adsorbed and intercalated water molecules; the second weight loss step (ca. 20%) occurs between 180 and 250 °C due to the thermal decomposition of HALS1 and HALS2 molecules; furthermore, the latter is confirmed by TGA curves of both neat HALS1 and HALS2, shown in the same figure; the third weight loss step (residual weight ca 55% and 60%, respectively), occurs between 250 and 700 °C due to dehydroxylation of the metal hydroxide layers and the loss of unexchanged nitrate ions.

However, as is already known, the stabilizing molecules show poor thermal stability at temperatures above ca. 200 °C and a tendency to volatilize, to migrate from the bulk to the surface of the manufacts, and to react with other chemicals during both manufacturing and service [23]. Recently, to avoid these inconveniences, the anchoring of the stabilizing molecules onto the surface of some inorganic particles, such as montmorillonite, layered double hydroxides, silica, etc., has been proposed. This approach can be considered an appropriate strategy for improving the thermal stability; for reducing the tendency to volatilize; and for preventing the migration of the stabilizing molecules. Therefore, the anchoring of the stabilizing molecules onto particles can be performed by both chemical covalent linkages and/or physical adsorption; thereby, preserving their stabilizing functionalities [20,23,24].

Although the exchange of LDH-NO_3_ by both HALS1 and HALS2 occurred partially, the obtained results could be considered satisfactory. This is because anchoring and adsorbing HALS1 and HALS2 onto the LDH-NO_3_ outer surface, and intercalating them between ceramic layers, offers the opportunity for these molecules to act selectively at the interface between inorganic structures and the organic host matrix. The organic/inorganic interface zone is critical to the beginning of composite degradation due to stress concentration; hence, the availability of stabilizing molecules in this zone could offer appropriate protection of the composite materials.

### 3.2. Characterization of PA11 Nanocomposites Containing LDH/HALS

Both novel LDH-HALS1 and LDH-HALS2 were incorporated by melt mixing in a PA11 matrix and the properties and performance of formulated PA11/LDH-HALS1 and PA11/LDH-HALS2 were investigated using morphological analysis, i.e., SEM and XRD analyses; rheological analysis; and through differential scanning calorimetry. Unfortunately, the TEM observation of these samples did not reveal any significant information about the LDH morphology; for this reason, it is not reported. In addition, a sample containing LDH-NO_3_ and commercial HALS (Cyasorb^®^ UV-3853; added at 1 wt%) was produced by melt mixing and investigated using the same characterization techniques for comparison.

**Figure 5:**
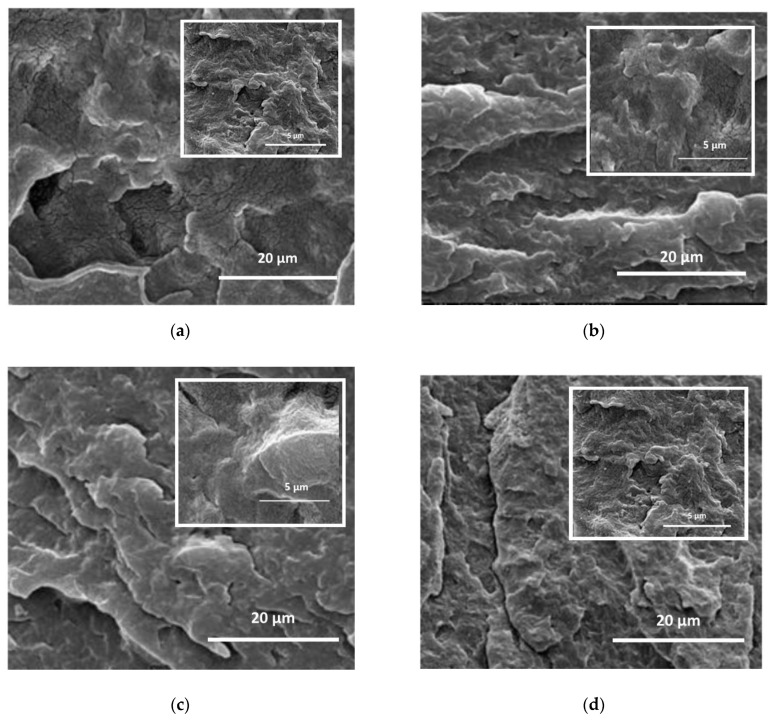
SEM images at different magnifications of (**a**) PA11/LDH-NO3, (**b**) PA11/LDH-HALS1, (**c**) PA11/LDH-HALS2 and (**d**) PA11/LDH-HALS_added_.

In Figure 5a–d, the morphologies of all investigated PA11-based nanocomposites are shown. It is important to highlight that, at these SEM magnifications, the overall morphologies of PA11-based samples are well noticeable, while the single LDH nanoparticles are not observable even at higher magnifications. It is worth noting that the PA11/LDH sample, i.e., without stabilizer, appears rough and large holes are noticeable, see Figure 5a. The morphologies of PA11-based nanocomposites containing LDH/HALS1, LDH/HALS2, and LDH/HALS_added_, i.e., containing free added HALS, appear smoother and no holes are visible at the considered magnifications (see Figure 5b–d); thereby highlighting the beneficial effect from the presence of stabilizing molecules. This is consistent with other analyses.

**Figure 6:**
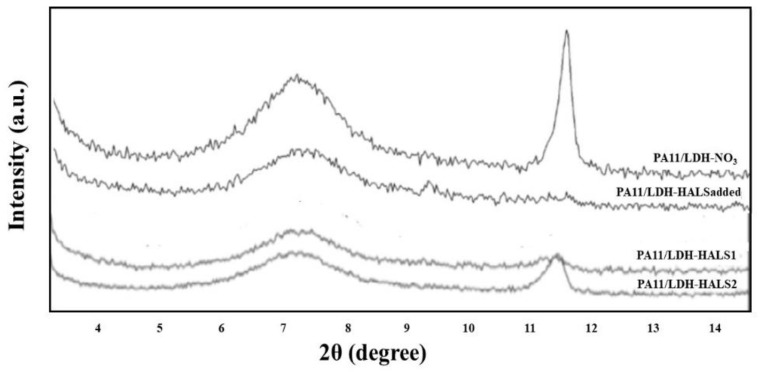
XRD patterns of all investigated PA11-based nanocomposites.

In Figure 6, the XDR traces of all investigated PA11-based nanocomposites are plotted. The PA11-based samples containing LDH-NO_3_ and LDH/HALS_addded_ show two well distinguished peaks, i.e., one broad peak at ca. 7° and one at ca. 11.2°, in the range 2*θ* = 1.5–12°, due to ceramic layered structures. It can be observed that these two peaks appear slightly pronounced in the XRD traces of both the PA11/LDH-HALS1 and PA11/LDH-HALS2 samples, thereby highlighting the existence of disordered LDH structures. This result suggests a beneficial effect on the nanocomposite morphologies of both HALS1 and HALS2 molecules that are anchored onto and between the LDH layers.

**Figure 7:**
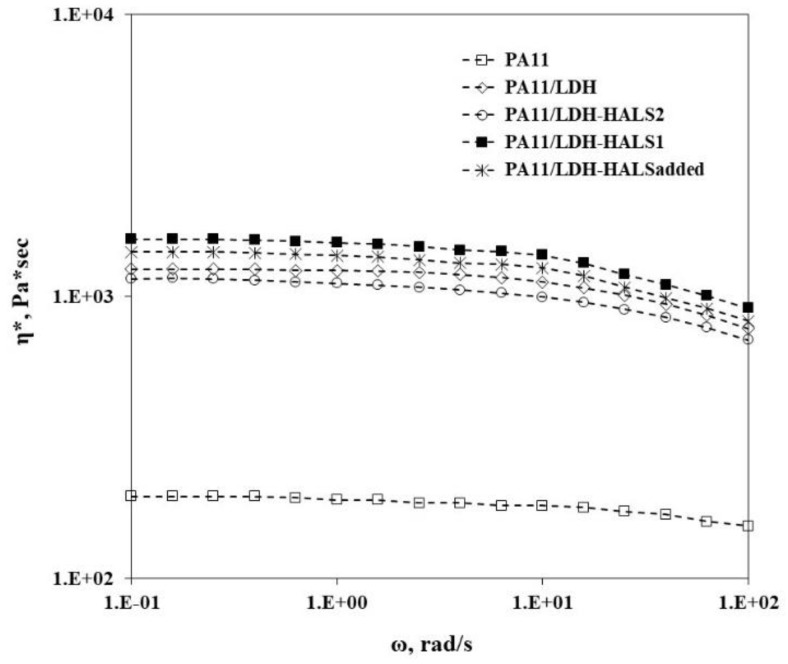
Viscosity curves (η*) of neat PA11 and all investigated PA11-based nanocomposites.

In Figure 7, the viscosity curves of neat PA11 and all investigated PA11-based nanocomposites are plotted. The viscosity values of all nanocomposites are significantly higher (more than one decade) at both low and high frequency ranges than the values of neat PA11. The latter can be understood by considering both the reinforcement effect of LDH, which is more pronounced in melt state at low frequencies, and the achieved good dispersion and distribution of LDH into the PA11 matrix, which is also consistent with the morphological analysis. It is worth noting that the samples containing HALS molecules show even higher viscosity values, again highlighting the beneficial effect of the stabilizing molecules.

**Figure 8:**
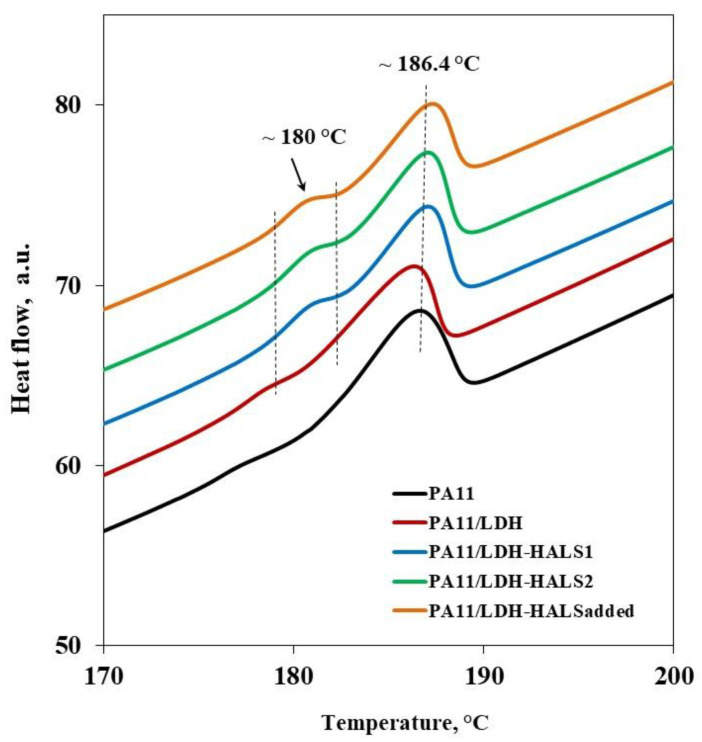
DSC curves (second heating scan) of neat PA11 and all investigated PA11-based nanocomposites.

Figure 8 and Table 1 show the results from the DSC analysis of neat PA11 and all investigated PA11-based nanocomposites. Neat PA11 shows a clearly visible main fusion peak at ca. 186.4 °C due to the presence of the α-crystalline form, and a shoulder at ca. 180 °C due to the presence of the γ-crystalline form, in accordance with the literature [6]. As can be seen, the presence of LDH makes the shoulder at ca. 180 °C more pronounced, suggesting the incrementation of the PA11 γ-crystalline form; this effect was documented in the literature for other ceramic nanoparticles such as montmorillonite [6,27]. As is shown in Table 1, due to the presence of LDH both Tm1 and Tm2 are slightly lower in comparison to the melting temperatures of neat PA11, and the results are slightly higher due to the presence of all kinds of LDH-HALS. Considering the values of fusion enthalpy from the first heating scan, it seems that unmodified and modified LDH exert a slight nucleating effect that is not noticeable in the second heating scan. This is because the LDH presence limits the macromolecules’ organization in regular structures., The short experimental time should also be taken into consideration here.

Therefore, in the well-known “shish-kebab” structure of a polyamide system, the “shish” structures are mainly based on γ-crystalline form, while the “kebab” structures are based on α-crystalline form. The presence of clearly noticeable shoulders in the DSC trace of nanocomposites is most likely due to an increase of the PA11 γ-crystalline form caused by the well dispersed LDH nanoparticles and free added HALS molecules hindering the growth of “kebab” structures; instead, favouring the formation of “shish” structures.

### 3.3. Photo-Oxidation Behaviour of PA11 Nanocomposites Containing LDH/HALS

To investigate the photo-oxidative resistance of ecofriendly PA11-based nanocomposite films, thin films of these materials were subjected to UVB exposure (using UVB lamps, having a broad emission in the range 280–330 nm with emission peak at 313 nm). It is worth noting that the overall aging time is accelerated more under UV exposure from UVB lamps than under exposure from other UV-sources, such as UVA, xenon, etc. Here, all of the films were exposed under the same conditions to aid comparison.

According to the literature, the predominant degradation pathway of polyamides in the presence of humidity is through the hydrolysis of amide bonds, while upon thermo- and photo- exposure, the predominant degradation pathway is through random chain scission. This leads to the formation of chemical defects, e.g., new additional carbonyl functions [6,21]. It was documented that the polyamide photodegradation can be profitable giventhe appearance of a peak at 1715 cm^−1^ in FTIR spectra due to formation of new additional carboxyl-containing functions.

**Figure 9:**
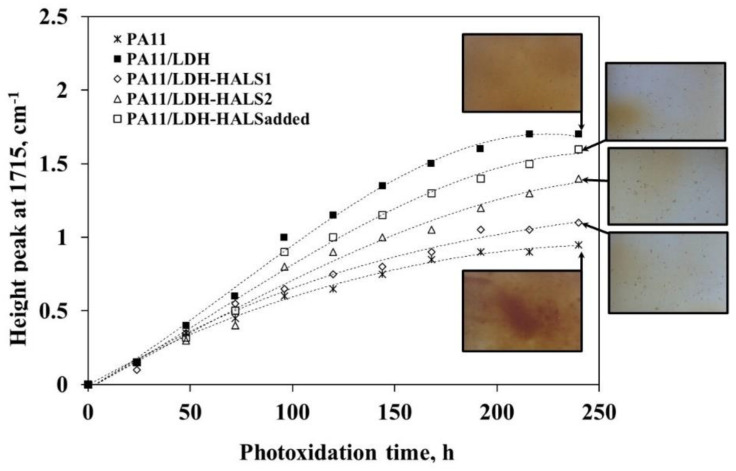
Height peak at 1715 cm^−1^ for neat PA11 and PA11-based nanocomposite films as a function of photo-oxidation time.

Figure 9 shows the increace in peak height of 1715 cm^−1^ as a function of photo-oxidation time of neat PA11 and all investigated PA11-based nanocomposites, while all acquired FTIR spectra are reported as Supplementary Information, see Figures S1–S5. The presence of LDH in PA11 causes a significant increase to the peak high of 1715 cm^−1^ in comparison to neat PA11 at same exposure time. Interestingly, this peak is less pronounced for samples containing LDH/HALS_added_, and even more for samples containing both LDH-HALS1 and LDH-HALS2. As expected, the presence of free added HALS molecules, especially anchored HALS1 and HALS2, cause the appearance of a less pronounced peak of 1715 cm^−1^; thereby, suggesting an improved photo-oxidative resistance in these nanocomposite films. Further confirmation comes from the visual inspections of all investigated samples at maximum exposure time, see images reported in Figure 9. Neat PA11 and PA11/LDH show significant yellowing (yellow to brown colour), while the samples containing stabilizing molecules appear to show only small yellow spots.

Therefore, the HALS molecules anchored onto the LDH structure are able to act in situ, i.e., at the interface between host matrix and inorganic particles, which is the critical site for the beginning of nanocomposite degradation. The delivery of stabilizing molecules in situ, i.e., at the interface between polymer/inorganic particles, offers the possibility to formulate ecofriendly biopolymer-based nanocomposites with significantly improved oxidative resistance. This is an innovative strategy for the protection of nanocomposite materials containing inorganic nanoparticles.

## 4. Conclusions

In this study, innovative, LDH-HALS1 and LDH-HALS2 stabilized, ecofriendly, biopolymer-based nanocomposite films were successfully formulated and characterized. The effective formulation of LDH-HALS1 and LDH-HALS2 was established based on WAXD, FTIR, and TGA analysis. Then nanocomposites based on PA11 and both unmodified and modified LDH were formulated by melt mixing and investigated using morphological (SEM and XRD), rheological, and differential scanning calorimetry analyses. In addition, a PA11-based sample containing LDH and free added commercial HALS was also formulated for comparison. Based on the obtained results, it can be concluded that the LDH/HALS1 and LDH/HALS2 are dispersed and distributed very effectively in a PA11 matrix.

The photo-oxidative resistance of PA11-based nanocomposites containing innovative LDH-HALS1 and LDH-HALS2 were monitored and compared to that of nanocomposites containing LDH and LDH/HALS_added_. The obtained results suggest a beneficial effect from the presence of free added commercial HALS (i.e., LDH/HALS_added_) and even more from anchored HALS (i.e., LDH-HALS1 and LDH-HALS2). The HALS molecules anchored to LDH are available at the interface between inorganic particles and the host matrix, i.e., at the sites of degradation, and have clearly noticeable beneficial effects in the improvement of the photo-oxidative resistance of biopolymer-based nanocomposite films.

## Figures and Tables

**Table 1:** DSC data of neat PA11 and PA11-based nanocomposites.

Title 1	1st Heating		1st Cooling		2nd Heating		2nd Cooling	
	T_m_ (°C)	ΔH_m_ (J/g)	T_c_ (°C)	ΔH_c_ (J/g)	T_m_ (°C)	ΔH_m_ (J/g)	T_c_ (°C)	ΔH_c_ (J/g)
PA11	187.5	43.5	159.3	48.4	186.4	40.7	159.0	48.1
Pa11/LDH-NO_3_	186.3	47.3	159.3	48.8	185.1	36.5	160.1	51.8
PA11/LDH-HALS1	187.4	48.2	160.5	49.4	187.0	37.0	160.1	50.4
PA11/LDH-HALS2	187.2	45.6	161.3	48.5	187.3	37.7	161.0	48.6
PA11/LDH-HALS_added_	188.1	46.5	160.2	49.0	187.2	37.8	159.5	49.2

## Data Availability

Not applicable.

## Supplementary Materials

The following supporting information can be downloaded at: https://www.mdpi.com/article/10.3390/ma15165778/s1, Figures S1–S5: FTIR spectra collected at different exposure time of neat PA11, PA11/LDH, PA11/LDH-HALS1, PA11/LDH-HALS2 and PA11/LDH-HALS_added_.
