# Supplementary material for: Ecofriendly Biopolymer-Based Nanocomposite Films with Improved Photo-Oxidative Resistance
Source: Materials (Basel). 2022 Aug 21;15(16):5778. doi: 10.3390/ma15165778 (PMC9412501; doi:10.3390/ma15165778)
Supplement: Supplementary file 1 [file materials-15-05778-s001.zip › materials-1866051-supplementary.pdf]

Supplementari Information

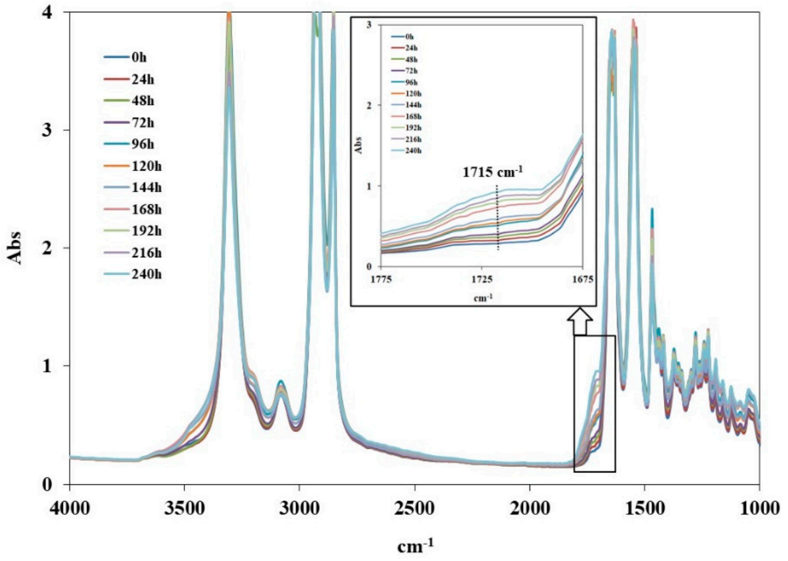

Figure S1. FTIR spectra collected at different exposure time of neat PA11

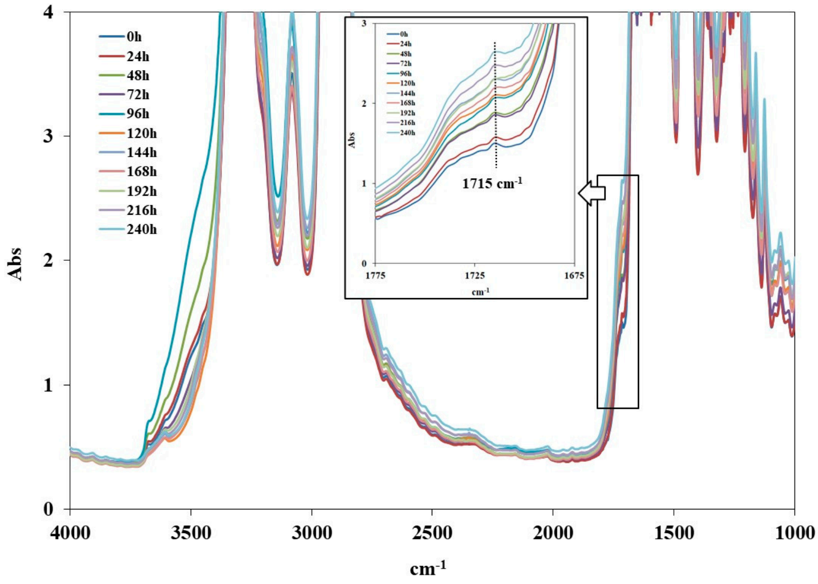

Figure S2. FTIR spectra collected at different exposure time of PA11/LDH

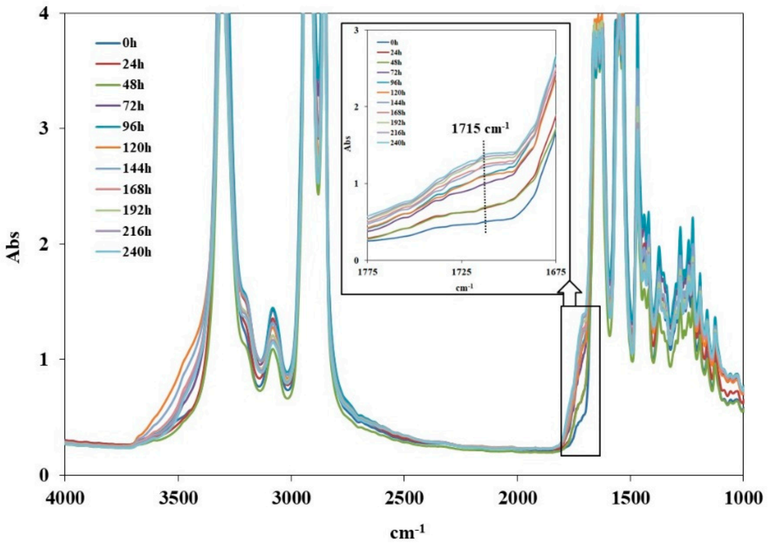

Figure S3. FTIR spectra collected at different exposure time of PA11/LDH-HALS1

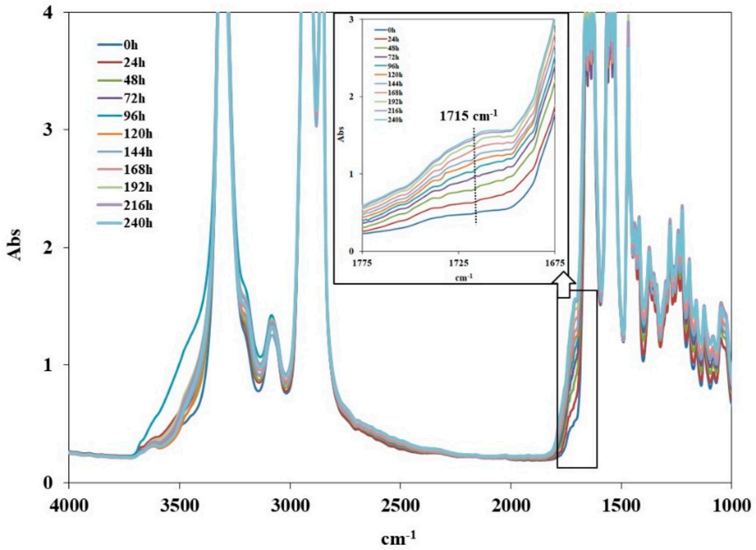

Figure S4. FTIR spectra collected at different exposure time of PA11/LDH-HALS2

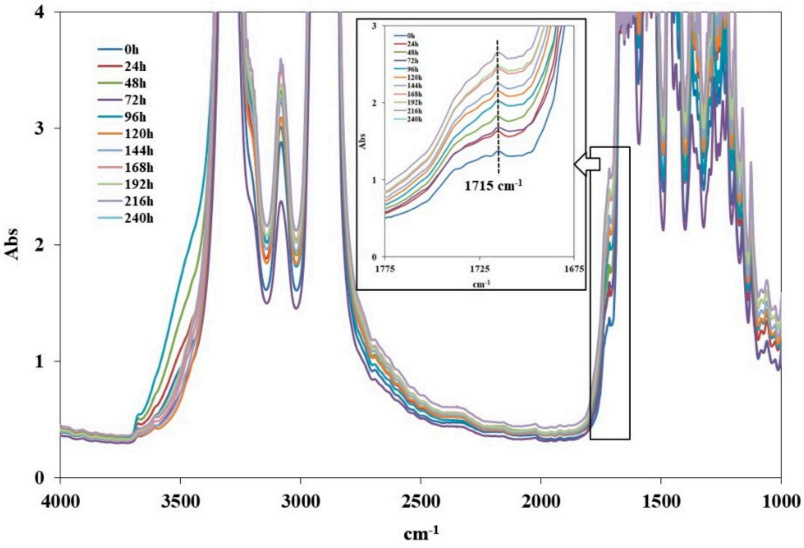

Figure S5. FTIR spectra collected at different exposure time of PA11/LDH-HALS$_{added}$
